# Musculoskeletal disorders among dental students: a survey from Saudi Arabia

**DOI:** 10.1186/s12903-023-03469-y

**Published:** 2023-10-25

**Authors:** Jood AlSahiem, Sarah Alghamdi, Raghad AlQahtani, Leena Bin-Jardan, Dalal AlMadani, Faraz Ahmed Farooqi, Balgis Gaffar

**Affiliations:** 1 College of Dentistry, Imam Abdulrahman Bin Faisal University, Dammam, Saudi Arabia; 2 Department of Dental Education, College of Dentistry, Imam Abdulrahman Bin Faisal University, Dammam, Saudi Arabia; 3 Department of Preventive Dental Sciences, College of Dentistry, Imam Abdulrahman Bin Faisal University, Dammam Costal Street, B.O Box 1982, 31441 Dammam, Saudi Arabia

**Keywords:** Musculoskeletal disorders, Dentist, Dental student, MSD, Musculoskeletal injures, Work related Musculoskeletal disorders, WMSD

## Abstract

**Background:**

Work-related musculoskeletal disorders (WMSD) are injuries affecting bones, joints, muscles, and tendons due to improper working conditions with serious consequences on health and career. Dentists were found to be at greater risk of developing WMSD compared to other healthcare providers. This study aimed to investigate the prevalence and risk factors of WMSD among dental students in Saudi Arabia.

**Methods:**

This cross-sectional survey-based study recruited dental students across the kingdom using respondent-driven sampling technique. Data was collected using the Nordic Musculoskeletal scale and a validated, self- administered, close-ended questionnaire which assessed WMSDs predisposing factors, enabling factors and musculoskeletal disorders consequences and management. The questionnaire was administered in both Arabic and English languages and was distributed online using google forms. Data analysis was performed using SPSS, Chi-square test or Fisher Exact test was used where appropriate and Multivariate Logistic regression analysis was performed to identify predictors of developing WMSDs.

**Results:**

The prevalence of WMSD was 87% (95% CL; 83.9% to 90.3%) among the 462 respondents. Gender, study year, type of practice, having clinics for left-handed, hours of clinical practice, sitting in the proper position while working, use of coping strategies were significantly associated with WMSD prevalence (*P* < 0.05). Males were OR = 10 times at higher risk of WMSD compared to females (*P* = 0.0001). Those with daily clinical practice were OR = 5 times at higher risk of WMSD than those who have weekly practice. Those practicing walking, workout, and yoga showed lower prevalence of WMSD (OR = 0.377 & 0.323, *p* = 0.015, 0.010 respectively).

**Conclusions:**

The prevalence of WMSD among dental students in KSA was high. Males and those with prolonged clinical sessions were at greater risk of WMSD. There is a need for awareness campaigns to educate dental students about risk factors of WMSD. Collegesy, dental colleges should adopt policies in reducing WMSD among their students.

**Supplementary Information:**

The online version contains supplementary material available at 10.1186/s12903-023-03469-y.

## Background

Musculoskeletal disorders (MSDs) are defined as injuries of the soft tissue caused by prolonged or impulsive, awkward body posture, and using repetitive forceful movements combined with an absence of recovery breaks or exercises [1–3]. These regions involve nerves, tendons, muscles, joints, and the cartilage in the extremities, neck, and lower spine [4]. Symptoms of WMSDs include pain, numbness, stiffness, puffiness, tenderness and/or weakness [4]. On the other hand, some of the most common signs include reduced grip, reduced range of motion in addition to loss of coordination [5]. Many case-definitions exist for WMSDs leading to variations in the prevalence and in the evaluation of risk factors. However, majority of WMSDs cases are diagnosed based on clinical signs and symptoms, physical examination, and imaging in addition to investigating work-related criteria [6].

WMSDs have profound consequences on individual’s health, wellness, and career worldwide [7]. Proposed risk factors include the usage of vibrating tools with high frequency, overstrained different postures, excessive movement of body joints, standing for a long duration and psychological stress [8, 9], with most of the WMSD resulting from overstrained repeated movements and few may arise from injuries [4]. Also, staying for a prolonged period of time at same position, either standing or sitting was listed as a major risk of MSD’s [10]. Vibrating tools are devices that transmit impulses of varying intensity (and are frequently used by the dentists, mainly the handpiece) are specifically linked to hand-arm vibration syndrome a syndrome with vascular and neurological changes affecting hand and fingers [11]. Dentists are the most susceptible healthcare providers for work-related musculoskeletal disorders (WMSD) being exposed to many of the previously mentioned predisposing factors [12].

During the last decade, several studies investigated the prevalence of WMSD among practicing dentists in Australia, Brazil, China, Czech Republic, India, Iran, Pakistan, Spain, Taiwan, Turkey, Lebanon, and UK [3, 10, 13–15]. From all MSD affected body areas, neck pain was the most reported [8]. Four studies conducted in Saudi Arabian cities of Hail, Riyadh, Dhahran, and Jeddah have highlighted the high prevalence of MSD and its effects on individuals' daily life [1, 3, 4, 8]. Alzayani and colleagues reported a high prevalence (72.6%) of WMSD and found that Saudi nationals, resident dentists, those with working experience of more than 5 years as well as those working more than 12 h per day were at greater risk of developing WMSD [1]. Almost similar prevalence (70%) was reported from Jeddah [3] and the authors linked increased risk of WMSD to female gender and lack of exercise. In Hail the prevalence of WMSD was 77.9% however they did not report any gender differences as a risk of WMSD [4]. A study among dental professionals found the prevalence of WMSD to be 85% [9]. Age, gender, working hours, speciality as well as the type of dentist’s chair were the reported factors associated with increased risk of WMSD [9].

Many preventive measures can be applied to reduce the risk of musculoskeletal disorders [12]. One of the approaches to alleviate the occurrence of musculoskeletal disorders among dentists is the use of magnification devices, such as dental loupes (a magnification glasses worn by dentists to improve visibility and reduce inappropriate neck bending), surgical microscope, and endoscopes [15]. Such devices magnify and allow better visualization of the working area, with adjustable working distance that follows the operator’s physical characteristics to improve ergonomics [16, 17]. In addition, to ergonomic saddle chair encourages the adoption of the spine to the correct posture which is the ‘s’ curvature [16, 17]. Regular exercises and frequent chair-side stretching may also help in preventing and reducing the incidence of musculoskeletal disorders by enhancing the blood flow, thus, increasing the synovial fluid in the joints, and provide efficient motion range [18].

Research suggests that WMSD is a major contributor to sick leave, reduced productivity, and may lead to early retirement and reduced quality of life [3, 4, 9, 19, 20]. WMSD was also linked to increased incidence of medical errors, exposure injuries, and disabilities on the long term[20]. Moreover, the socioeconomic impact of WMSD is considerable, resulting in substantial consumption of healthcare resources, absenteeism from work, disability, and compensation payments [20, 21]. Previous studies in the Kingdom of Saudi Arabia (KSA) were limited to specific cities which may not allow a complete understanding of the predisposing and alleviating factors of WMSD. Most of them included smaller sample size which might have also hindered the generalizability of the findings. Most importantly, there is lack of information on the prevalence, severity, and risk factors of WMSD among dental students. Given that previous studies have linked the prevalence and severity of WMSD to the duration and extent of exposure to the risk factors [1, 3–14], it is therefore crucial to investigate the onset of WMSD at early stages of clinical practice. This will help in early detection as such preventing long term consequences as well as will allow prioritizing preventive interventions and policies within dental schools. Therefore, the purpose of this study was to investigate prevalence of WMSD and to define the possible risk factors among dental students in Saudi Arabia.

## Materials and methods

### Study design and setting

This cross-sectional, survey-based study was conducted during the period from December 2021 to January 2022 and included a sample representing dental colleges in the North, East, West, South and Middle Provinces in Saudi Arabia.

### Study participants

There are 26 dental colleges in Saudi Arabia (the majority are public), with almost 100% Saudi nationals in public schools (with some exceptions for refugees) while private schools accept other nationalities. Female students are slightly fewer than the male students and the age of admission to first year in the university (health track) ranges from 18 to 20 years old. The study included dental students from third to sixth year as well as interns studying in any dental college in Saudi Arabia and who agreed to participate in the study. Those with any previous medical conditions (such as diabetes, hypertension, endocrine or hormonal disturbances) or currently under medical treatment (that requires taken anti-inflammatory or painkillers, or has side effects on joints/muscles/bones) were excluded from the study.

### Sample size and sampling technique

Participants were recruited through respondent driven sampling. It is a type of snowball sampling technique where the participants recruit their peers who are part of their social network, who in turn become recruiters [22]. This sampling technique is usually used when it is difficult to reach out to participants. The participants in the current study were dental students from all over the kingdom. It was difficult to reach them in person. Snowball sampling technique was proven effective and can encourage participants (being unknown to the researchers) to share real and honest data [22] This sampling technique was also proven efficient in reaching more participants in shorter period of time [23, 24].

Sample size was determined using an online calculator (https://sampsize.sourceforge.net/) [25] based on the formula for calculating adequate sample size for prevalence studies [26]. Prevalence was obtained from similar studies [3, 10, 13, 14] and an assumed prevalence of 0.6, a precision of 0.04 and a 95% level of confidence interval were considered yielding a total of 574 students. It was difficult to calculate a weighted sample as the number of dental colleges within each province differs, as well as the number of students in each college (which also differs within the same college depending on the annual acceptance rate) and most importantly, in some provinces there are newly established dental colleges which still do not have students in higher study levels. To compensate for the lack of weighted sample for each province we used raking and matching.

### Data collection tool

Data was collected using the Nordic Musculoskeletal scale and a self-structured questionnaire [27]. The questionnaire consisted of four parts: Demographics, daily habits and lifestyle, the Nordic Musculoskeletal scale, and questions about MSD management. The questionnaire was pilot tested on 20 dental students before the start of the study who were not part of the main study. The pilot testing results revealed very minor linguistic issues, which were corrected. As a result, no major revisions were made, and no interpretation requests were received. The questionnaire was available on both Arabic and English languages and distributed online using online google forms. The Arabic version of the questionnaire was validated by five content experts with the response sheet about the relevance of each item and the score of 0.87 of content validity index (CVI) was considered as acceptable/good. To measure the content validity ratio (CVR) the questionnaire was given to 10 health experts working in the public sectors (dentists, physicians, and physical therapists) to assess the necessity of each item of questionnaire. None of the items received less than 0.7 score. Furthermore, Cronbach alpha was used to assess the instrument's reliability, and a value of (0.83) was considered good consistency.

### Predisposing (Personal) factors

This section collected demographic data of the participants namely: the age of the student in years, the gender of the student (male or female), region (Eastern, Central, Western, Northern and Southern), study year (Third, Fourth, Fifth, Sixth and interns), type of school (Public or Private), type of practice (Clinical, pre-clinical or both), in addition to student’s height and weight mentioned in centimeters and kilograms respectively. Participants were also asked about their dominant hand, are they right or left-handed.

### Enabling factors

Information about the daily habits and lifestyle of students was collected through 12 close-ended questions. A question if there was a prepared clinic/station for left-handed students in their dental school answered as yes or no. Number of clinical practice hours categorized as daily or weekly. The average time of clinical/pre-clinical activity per day categorized as between 2 to 4 h, 5 to 7 h, or between 8 to 10 h. Participants were also asked about the session they feel most exhausted in, and could choose one of the following specialties Endodontic, Restorative, Fixed prosthodontics, Removable prosthodontics, Periodontics, Pedodontics, Oral and Maxillofacial surgery, Orthodontics or Radiology. Participants were asked if they use dental magnification loupes with possible answers of yes, no, or sometimes; and if they choose the appropriate gloves size answered as yes, no or I do not care; and if they consider the principles of ergonomics while working answered as yes, no or sometimes. Students were asked about how many hours per day do you use electronic devices (mobile phones, tablets, laptops and portable electronic games) and pick 2 to 4 h/day, 4 to 6 h/day, 6 to 8 h/day, or more than 8 h/day). Similarly, how many hours on average do they sleep daily and can choose less than 6 h or more than 6 h per day. Students’ preference in working if they prefer working in a standing or in a sitting position were also investigated.

### Alleviating factors

The daily life practices of the students were investigated through a single close-ended question namely if they practice one, some, or all of the following: working out, walking, yoga, meditation and/or stretching exercises.

### Prevalence of musculoskeletal disorders

Nordic Musculoskeletal scale was used to estimate the prevalence of WMSD among the participants and to identify its severity per body site. This is a valid and reliable scale that originally included 40 close-ended questions aided by a body map to identify the body sites affected by WMSD along with questions about symptoms, incidents leading to the condition along with questions assessing pain and life restrictions in past 12 months [4, 20]. The scale includes 2 questions 1) to specify the location of the pain, 2) if they had any musculoskeletal problems in the last 12 months answered as yes or no; and if yes to specify the body parts with pain and they got to choose one or all of the following:neck, shoulders, elbows, wrists/hands, upper back, lower back, hips/thighs, ankles, feet, or not applicable if they have no WMSD pain [4, 20].

### Musculoskeletal disorders consequences and management

Participants were asked how they manage WMSD pain with options such as pain killers, hot bath, cupping therapy, stretching exercises or not applicable. We also asked if participants think that working with an assistant will improve their WMSD condition answered as yes, no, or not applicable. Participants were asked if they give sufficient attention to their health, and they respond by either yes or no. Lastly, students were asked if they believe that WMSD pain affects their life and they got to choose either yes, no, or not applicable.

### Data collection procedure

The online questionnaire was created using Google forms and QR code was generated and shared with students in dental colleges using social media namely Twitter, WhatsApp, and Facebook. The QR code was also circulated by participants to their friends and colleagues within each college. A list of the 26 dental schools in the kingdom was prepared by the research team. A network search was carried out to identify students who are leaders of students’ activities within each dental school. Dental students were approached by the research team during social events, students’ clubs, and students’ platforms. An introduction preceded the survey explaining the purpose and eligibility criteria and a request to share the link with their network. Each participant could only complete a single questionnaire through IP address restrictions. The survey was kept active for the period of the study (The survey was active from 8th of December 2021 to 8th of January 2022) after which no more responses were accepted.

### Ethical considerations

The study was approved by the Institutional Review Board—Deanship of Scientific Research- Imam Abdulrahman bin Faisal University (IRB-2022–02-371). The survey was preceded by an explanation of the purpose of the study, the research team, and the time required to complete it. Participants were informed of the confidentiality and anonymity of their responses, as well as the importance of their voluntary participation. Participants have to tick a box “I have read the introduction and that choosing to proceed with the survey will be considered as a consent to participate in the study”, only upon ticking the box participants will be directed to the survey page.

### Statistical analysis

Descriptive statistics were calculated and presented as frequencies, percentages, means and standard deviations (SD) where appropriate. For association between qualitative variables Chi-square test or Fisher Exact test was implemented where appropriate. To identify predictors of developing musculoskeletal problems, a multivariate logistic regression analysis was carried out. All independent variables were introduced as predictors in multiple regression analysis, while demographics and medical condition remained constant. At first, univariate regression was used, with all demographic characteristics fed as independent variables and MSD prevalence as dependent variables. Following that, all demographic factors were entered into the multivariate model for combined effect. Data analysis was performed using SPSS, version 23.0 (IBM Corp., Armonk, NY, USA). Missing data was handled by assigning a certain code in the SPSS missing value cell during the analysis. *P*-values of < 0.05 were considered statistically significant.

## Results

Out of 574 invited, a total number of 426 participants agreed and returned a completed questionnaire contributing to a response rate of 74.2%. Mean age of the participants was 22.8 ± 2.45 years old and slightly more than half 221(52%) were males. Most students were from the Eastern region 220 (52%), studying in public schools 378 (86%) and from senior years. The majority of the students (242 = 57%) were less than 24 kg/m^2^. All demographic characteristics of the participants are presented in Table 1.

**Table 1:** Background information of the study participants

Demographic data	Number (%)
Gender	
Male	221 (51.9)
Female	205 (48.1)
Region	
Eastern	220 (51.6)
Central	204 (47.9)
Southern	2 (0.5)
Study Year	
Third year	64 (15.0)
Fourth year	70 (16.4)
Fifth year	77 (18.1)
Sixth year	126 (29.6)
Intern	89 (20.9)
School	
Public	378 (88.7)
Private	48 (11.3)
Type of practice	
Clinical	366 (85.9)
Pre- Clinical	49 (11.5)
Both	11 (2.6)
Age (in years) Mean(± SD)	22.8 ± 2.45
Height (in cm) Mean (± SD)	166.12 ± 9.1
Weight (in KG) Mean (± SD)	66.15 ± 14.7
BMI	23.8 ± 4.2

*SD* Standard deviation, *BMI* Body mass index

**Fig. 1:**
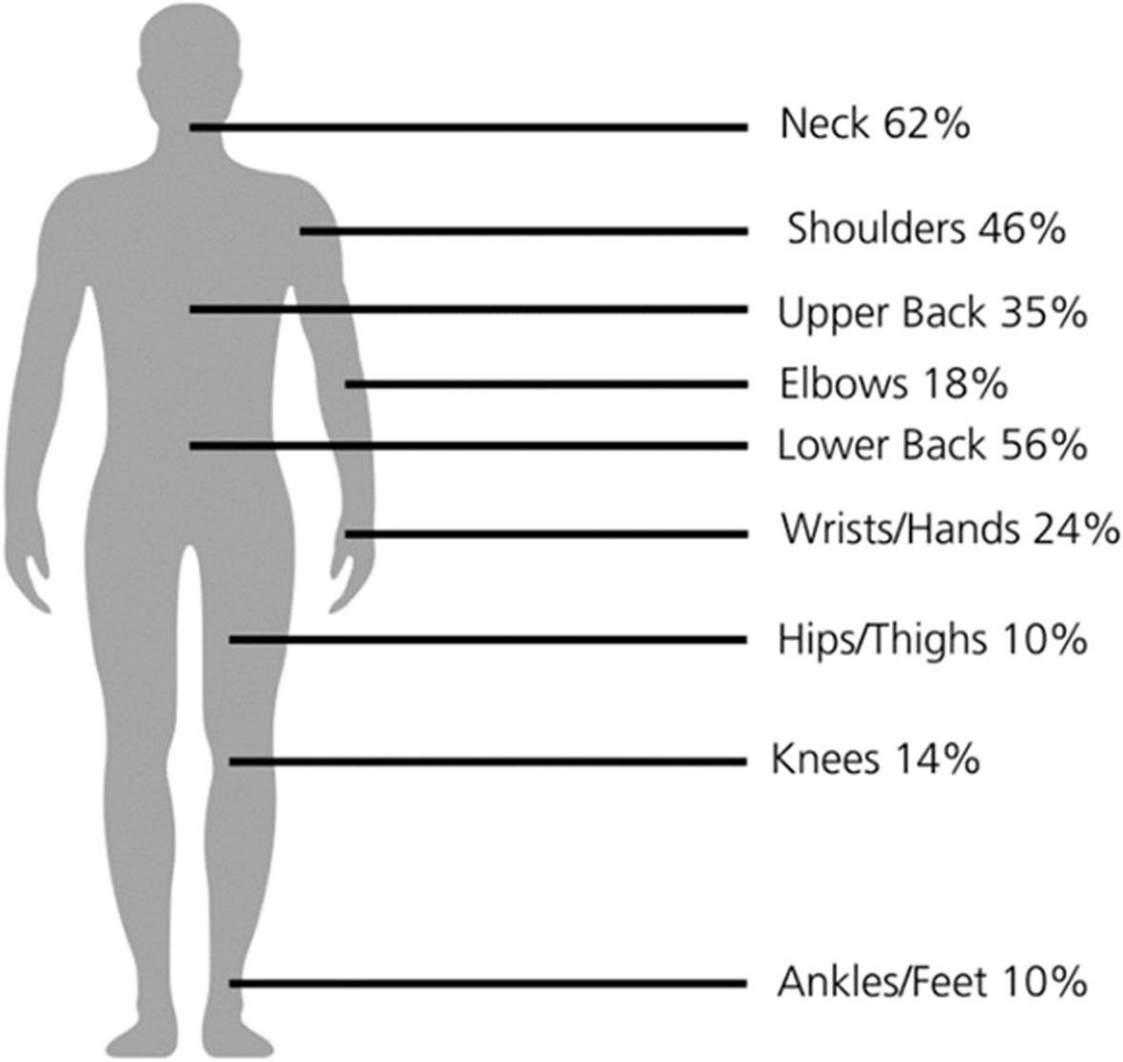
Prevalence of MSD on different body sites

Among the 426, 87% (95% CL; 83.9% to 90.3%) of respondents reported having WMSD on at least one side of their body. Figure 1 shows that the highest prevalence of WMSD pain was reported in the neck (62%) followed by lower back (56%) and shoulder (46%) whereas the lowest prevalence was reported in hips/thighs (10%) and ankles/feet (10%).

Table 2 presents the comparison between gender and daily habits and lifestyle of the respondents. There were more males who are lefthanded than female (8% vs 4%), moreover both genders reported having assigned clinics designated for left-handed persons. Practice hours daily and weekly for both male and female was almost similar, and the average clinical duration was also similar 2–4 h for the majority. No association was found between the gender of the student and practice hours or average clinical time (p-value 0.101, 0.12 respectively). Females were more likely to sit in the correct position compared to male participants (58% vs 46%), similarly females were more likely to workout (47% vs 17%) with a statistically significant difference p-value = 0.012 and 0.001 respectively. Females were more likely to spend more time (6–8 h) on electronic devices compared to males (34% vs 26%, respectively) similarly, females were more likely to sleep for more than 6 h than their counterpart (58% vs 48%) a difference that was statistically significant (P-value 0.042,0.036 respectively).

**Table 2:** Differences in daily habits and lifestyle between both genders

Daily habits and lifestyle	Gender		*P*-value
	Male n(%)	Female n(%)	
Dominant hand			
Right-hand	204 (92.3)	197 (96.1)	0.072
Left-hand	17 (7.7)	8 (3.9)	
Is there a prepared clinic for left-handed students in your dental school?			
Yes	131 (59.3)	125 (61)	0.400
No	45 (20.4)	32 (15.6)	
I Don’t Know	45 (20.4)	48 (23.4)	
Practice hours			
Daily	187 (84.6)	183 (89.3)	0.101
Weekly	34 (15.4)	22 (10.7)	
Average time of clinical\pre-clinical activities per day			
2–4 h	108 (48.9)	101 (49.3)	0.12
5–7 h	84 (38)	89 (43.4)	
8–10 h	29 (13.1)	15 (7.3)	
Use of dental magnification loupes			
Yes	50 (22.6)	43 (21)	0.385
No	171 (77.4)	162 (79)	
Choosing appropriate gloves size carefully			
Yes	167 (75.6)	164 (80)	0.284
No	54 (24.4)	41 (19.5)	
Sitting in the correct position in the clinic and lab			
Yes	93 (46.5)	115 (58.4)	0.012*
No	107 (53.5)	82 (41.6)	
Current physical practices			
Workout	38 (17.8)	96 (47.3)	0.001*
Walking	65 (30.5)	34 (16.7)	
Yoga	16 (3.8)	5 (1.5)	
None of the above	102 (47.9)	70 (34.5)	
Use the electronic devices per day			
2-4 h	96 (43.4)	90 (43.9)	0.042*
6-8 h	57 (25.8)	70 (34.1)	
More than 8	68 (30.8)	45 (22)	
Hours of sleep on daily average			
Less than 6	114 (51.6)	87 (42.4)	0.036*
More than 6	107 (48.4)	118 (57.6)	
Work preference			
Standing	167 (75.6)	167 (81.5)	0.21
Sitting	54 (21.7)	38 (17.6)	
Belief that the pain affects the daily life			
Yes	178 (80.5)	151 (73.7)	0.218
No	21 (9.5)	24 (11.7)	
NA	22 (10)	30 (14.6)	
Working with assistant can improve MSD condition			
Yes	193 (87.3)	183 (89.3)	0.712
No	28 (5)	22 (3.4)	
Give sufficient attention health			
Yes	66 (29.9)	101 (49.3)	0.0001*
No	155 (70.1)	104 (50.7)	

^*^Statistical analysis using Chi-square test

Table 3 shows the association between prevalence of WMSD and daily habits and lifestyle. Gender, study year, type of practice, having clinics for left-handed, hours of clinical practice, sitting in the proper position while working, use of coping strategies were significantly associated with WMSD prevalence (*P* < 0.05).

**Table 3:** Factors associated with increased risk of MSD among the study participants

Factors	Musculo- Skeletal pain						
	Variables	*p*-value	OR	95% CI	*p*-value	Adjusted OR	95% CI
Predisposing factors	**Age**	0.998	0.966	0.889–1.12	0.328	1.136	0.879–1.468
	**BMI**	BMI	0.099	0.947			
	**Gender**						
	Female	ref					
	Male	**0.001**	3.329	1.777–6.237	0.0001*	10.082	3.192–82.416
	**Dominant hand**						
	Left-hand	ref					
	Right-Hand	0.636	1.307	0.431–3.962	0.737	1.38	0.21–9.05
	**Study Year**						
	Third year	ref	1				
	Fourth year	0.81	0.899	0.81–0.899	0.804	1.326	0.144–12.244
	Fifth year	0.015*	12.88	0.015–12.88	0.133	7.58	0.538–106.7
	Sixth year	0.979	1.011	0.979–1.011	0.86	1.14	0.265–4.898
	Intern	0.646	1.197	0.646–1.197	0.291	1.946	0.241–3.242
	**Type of practice**						
	Pre- Clinical	ref					
	Clinical	0.462	0.735	0.324–1.668	0.380	2.893	0.271–30.937
	Both	0.044*	1.979	1.019–3.844	0.347	1.731	0.551–5.433
	**School**						
	Private	ref					
	Public	0.321	0.583	0.201–1.691	0.853	0.885	0.241–3.242
Enabling factors	**Clinics for left-handed**						
	I Don't Know	ref					
	Yes	0.541	1.241	0.621–2.481	0.462	0.639	0.91–1.05
	No	0.038*	2.945	1.062–8.166	0.192	2.942	0.21–9.05
	**Practice hours**						
	Weekly	ref					
	Daily	0.045*	2.074	1.017–4.232	0.012*	5.084	1.42–18.17
	**Hours clinical\pre-clinical activities**						
	2–4 h	ref					
	5–7 h	0.74	1.106	0.61–2.005	0.538	0.539	0.075–3.853
	8–10 h	0.395	1.611	0.536–4.841	0.939	0.93	0.143–6.042
	**Sitting Correctly**						
	No	ref					
	Yes	0.045*	0.511	0.266–0.984	0.463	0.717	0.29–1.74
	**Hours of using Electronic Devices**						
	2-4h		1				
	6-8h	0.495	1.264	0.645–2.479	0.054	0.284	0.08–1.02
	More than 8	0.189	1.643	0.784–3.446	0.683	0.758	0.2–2.86
	**Average Daily hours of sleep**						
	Less than 6	ref					
	More than 6	0.783	1.083	0.613–1.912	0.705	0.851	0.37–1.96
	**Use Dental magnification loupes**						
	No	ref					
	Yes	0.728	0.888	0.455–1.735	0.364	1.689	0.54–5.24
	**Choosing appropriate gloves**						
	No	ref					
	Yes	0.26	0.648	0.305–1.378	0.927	0.951	0.32–2.82
	**Work Preference**						
	Standing	ref					
	Sitting	0.298	2.38	0.468–12.199	0.372	0.589	0.18–1.88
Alleviating factors	**Practicing preference**						
	None of the above	Ref					
	Workout	0.002*	0.312	0.151–0.644	0.015*	0.377	0.172–0.829
	Walking	0.034*	0.420	0.188–0.938	0.010*	0.323	0.136–0.766
	Yoga	0.672	0.713	0.148–3.427	0.55	0.554	0.101–3.028

^*^Showing statistical significance at 0.05

^*^Multivariate Logistic regression; Nagelkerke *R*^*2*^ = *0.55, Hosmer and lemeshow, p* = *0.62*

When combined all variables in multivariate logistics regression analysis male participants were 10.82 times at higher risk of WMSD (*p* = 0.001) compared to females. Those who reported daily clinical practice showed 5.084 times higher risk of WMSD than those with clinical activities scheduled weekly. While those who practiced walking or workout had lower odds of WMSD Table 3.

**Fig. 2:**
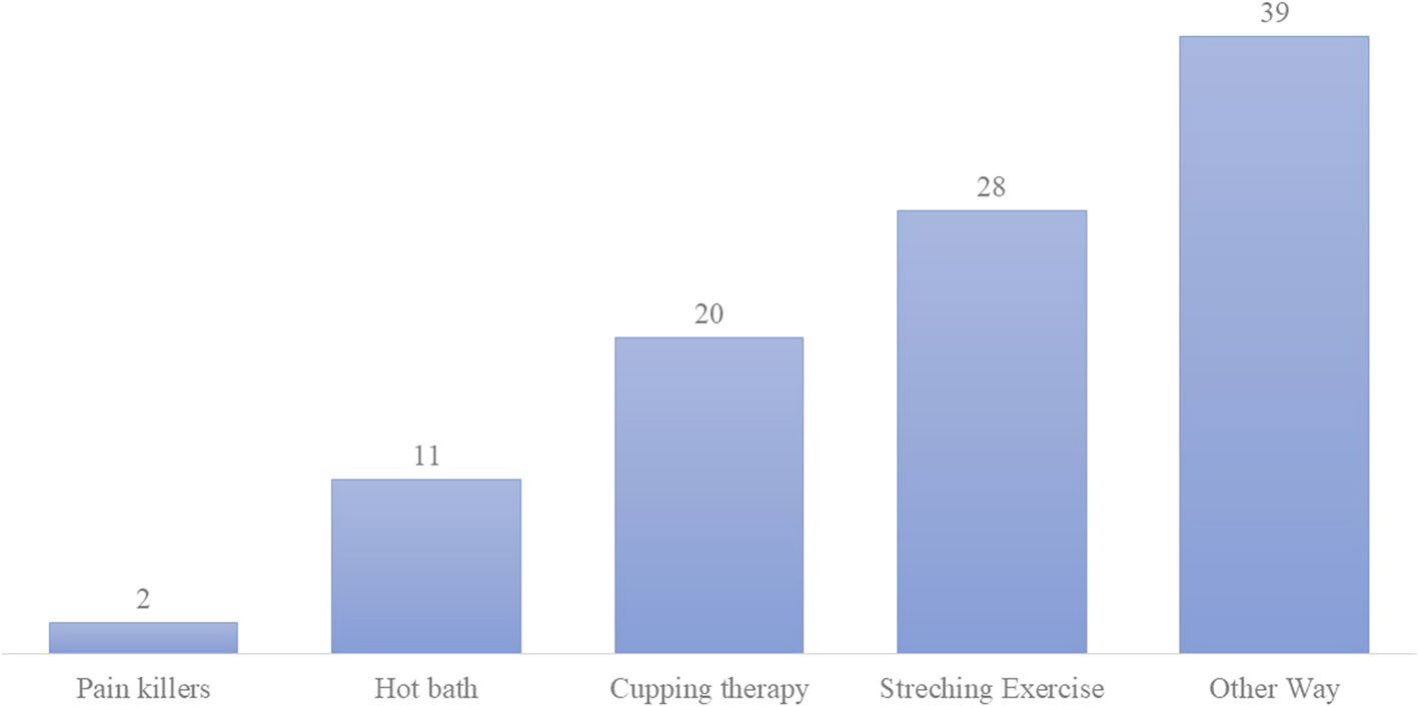
Methods/measures of managing MSD pain as reported by the study participants

Respondents complaining about WMSD pain during the last 12 months were more likely to believe that pain affects their daily life routine (82%) compared to those who did not complain from WMSD (*P* = 0.001). Figure 2 shows the different methods of managing WMSD pain as reported by dental students. Methods of managing WMSD pain were significantly associated with WMSD pain as well as believing that daily life is affected by the WMSD pain (*P* = 0.0001).

Table 4 shows factors that influenced the impact of WMSD pain as reported by dental students. Methods used by students to manage WMSD pain as well as the impact on daily life were significantly associated with WMSD pain. However, four handed dentistry (working with an assistant) was not associated with WMSD pain (*P* > 0.05).

**Table 4:** Factors affecting MSD pain as reported by dental students

Variables	MSD		*p*-value
	Yes	No	
Ways of managing MSD			
Pain killers	105 (28.3)	7 (12.7)	0.0001*
Hot bath	108 (29.1)	3 (5.5)	
Cupping therapy	9 (2.4)	0 (0)	
Not Applicable	117 (31.5)	43 (78.2)	
Other	32 (8.6)	2 (3.6)	
Working with a dental assistant			
Yes	331 (89.2)	45 (81.8)	0.09
No	40 (10.8)	10 (18.2)	
Impact on daily life			
Yes	304 (81.9)	25 (45.5)	
No	41 (11.1)	4 (7.3)	0.0001*
NA	26 (7)	26 (47.3)	

^*^Statistically significant at 0.05 from Chi-Squar Test/Fisher Exact

## Discussion

The prevalence of WMSD in at least one body site was 87% mainly in the neck. Males were 10 times at greater risk of WMSD females, similarly those who have daily clinical sessions were 5 times at greater risk of WMSD. WMSD is an accumulative condition and the severity of the symptoms, and the complications are proportional to the duration of exposure to risk factors. As such, dental students need to be educated about proper working conditions and ergonomic practices to prevent WMSD.

In consistence with similar studies nationally [1] and worldwide from Australia [8], Iran [10, 28], United Kingdom [14], and Germany [17], the prevalence of WMSD among dental care providers is extremely high. In the present study, 87% of participants reported having WMSD on at least one body site, this percentage is lower than studies done by Ohlendorf et al. [2] and Dajpratham et al. [29] and higher than others [1, 3, 4, 9, 13, 14, 30]. Musculoskeletal disorders are common work-related injuries especially among dental care providers with their onset usually starting during undergraduate studies and clinical training [18] and serious health [7, 20, 27] and economic consequences [31].

The lower back was the most affected body site in most studies [1, 3, 4, 8, 11, 28], which can be the result of insufficient lumber support when students sit on the dental chair [32]. In contrast, in our study the neck was the most affected body site by WMSD, this can be attributed to the decreased use of the dental loupes among dental students which were used by less than a quarter. Dental loupeswere proved efficient in reducing WMSD by improving work posture and preventing improper bending of neck [15–17]. Another factor that may have contributed to the neck being the most affected in the current study is the daily prolonged use of electronic devices. A recent study reported that the use of portable electronic devices (laptops, tablets, and phones) resulted in greater neck flexion increasing or exposing the individual to chronic neck pain [33]. Health promotion campaigns within colleges and in public should encourage students to reduce the use of electronic devices, at least during school time. Dental students may also consider replacing the use of tablets and laptops with desktop computers at home.

Although many studies reported females to be at higher risk of WMSD than males [8, 10, 12, 14, 17], in the current study we found that males were the ones with higher risk of WMSD. This might be due to the observed differences between both genders in their daily habits and lifestyle. As we found that females have better sleeping hours, ergonomics, as well as regular workout sessions during the week. As the association between WMSD and gender worldwide is controversial, Lima and Coelho suggested the need for gender specific preventive measures [34]. Also, gender differences should be interpreted considering physiological and social factors; married women may have extra tasks at home compared to those who are single as such leading to decreasing or eliminating the difference in WMSD risk between genders. Similarly, women at menopause would be at greater risk compared to younger women because of osteoporosis, osteoarthritis, and sarcopenia [35]. Therefore, there is a need for tailored preventive measures to address gender differences as well as differences between females themselves. It is also important to highlight the role of psychosocial factors in increasing the risk of WMSDs. These factors are individual’s subjective perceptions about work organization [36]. In the current study we assessed the difference of many psychosocial factors between genders. Females adopted more positive work practices such as sitting in the correct position in the clinic/lab, performed some physical activities after work, slept well on average, and gave sufficient attention to their health and all these factors were reflected well in lower incidence of WMSD among females.

Obesity and overweight increase the risk of osteoarthritis and that is why they are considered as risk factors for musculoskeletal disorders [37]. Onyemaechi et al. found that people with increased BMI have increased lumbosacral angles which make them at increased risk of MSD especially in the lower back [38]. Surprisingly, in the current study we did not find an association between BMI and WMSD. The incidence of obesity is decreasing in Saudi Arabia [39] and this can be due to adopting healthy dietary habits as well as regular physical activities leading to the observed diminished impact of obesity on WMSD in the current study. Improved lifestyle practices, healthy eating and regular exercising should be sustained in communities, especially among students to help in preventing both problems collectively.

A major factor that increased the risk of WMSD was daily clinical sessions in accordance with other studies [1, 8, 30]. This reflects the fact that dentistry is a challenging profession that requires an extra level of precision, compromised working positions as well as repetitive forceful movements [40]. Early clinical years and clinical work have been linked to increased prevalence of WMSD [41]. According to Meisha et al. [3] and Aboalshamat et al. [42], dental students’ awareness about WMSD was extremely low which is an alarming finding. Dental care providers in the early stage of their clinical practice and especially students may have difficulties in adopting proper grip and proper working positions. Therefore, it is highly recommended to include educational materials about WMSD in the curriculum and to provide awareness campaigns about WMSD for the health and wellbeing of dental students [19, 41]. Attention should also be given to clinical schedules to allow regular breaks between patients for stretching and exercising.

Although in the current study-and like previous work [43], no association was found between dominant hand and risk of WMSD, yet we observed that lacking prepared clinics for left-handed within the school increased the risk of WMSD by two folds. The difference between right and left-handed when it comes to WMSD was related only to the body site affected and the symptoms expressed [43] and not to prevalence of the condition. Left-handed suffer more from neck and shoulder pain compared to right-handed and as such may express different signs and symptoms. However, comparative studies did not find differences between left and right-handed in the prevalence of MSD [43].

In the present study, approximately more than half of the participants performed some physical exercises. This finding was supported by Dajpratham [29] who stated that most dental personnel perform exercise, although they do not work out regularly [32]. While some studies found that dental students are less likely to exercise [30, 34]. The differences observed between dentalcare providers in severity of WMSD have been linked to many factors with physical activity being of utmost importance [2, 8, 10, 28, 34, 44]. Regular exercises could help prevent the occurrence and reduce the intensity of WMSD [45, 46], as well as it can provide mental relaxation from the high stress that students encounter during their work and training [43] leading to a healthier lifestyle and decreased risk of WMSD [44]. Regardless of the type of occupation, regular exercising, workout, and yoga are crucial preventive measures against many chronic diseases [47]. Many colleges are implementing physical activity classes with their curriculum, this should be part of accreditation requirements to encourage all dental schools to do the same. It is also recommended to acknowledge gender differences when designing physical and sport activities.

In the current study the majority used stretching exercises to manage WMSD pain, however a greater portion mentioned using “other ways” for pain management. Therefore, focused group discussions and interviews are needed to identify what are the different management practices of WMSD among students and evaluate their appropriateness.

The findings of the current study shed light on WMSDs risk factors and risk groups among dental students. Musculoskeletal disorders have serious consequences on clinician’s health and career and the extent of these consequences are directly linked to the prolonged exposure to risk factors. Implementing preventive measures within dental schools such as ergonomic practices, design of dental clinics and laboratories, introducing physical education sessions and yoga classes are needed to help in controlling the long-term consequences and morbidities. It is necessary to conduct regular educational campaigns (especially among those starting their clinical sessions) to raise awarness about WMSDs and their risk factors among dental students. University and dental college administrations may develop policies that mandate annual medical checkups to detect and manage WMSDs at early stages. There is also a need to investigate ergonomic knowledge among dental students and the existence of ergonomics principles within dental curricula.

This study has some limitations, its cross-sectional design allows only for the establishment of possible association between WMSD and the investigated study variables. The usage of the online method of distribution may have led to non-response bias, therefore the generalizability of findings might be affected. The sampling technique used (respondent-driven) is also criticized for recruiting participants from social networks and not from the population frame which may have resulted in self-selection bias. When using a self-administered questionnaire, the responses of the participants might be affected by their personal situations and experiences and their answers may not reflect the real situation. Self-reported data may over or underestimate the investigated condition. Similarly, there is a risk of social desirability bias. Moreover, graduated dentists were not included in this study which may limit the establishment of an association between age/years of practice and WMSD. Within the highlighted limitations, this is the first study at a national level in the kingdom which allowed the study of different risk factors. The use of the validated Nordic questionnaire [43] ensures the reliability and the validity of the estimation of WMSD which in turn helps in comparing the current findings to previous work.

## Conclusion

The current study shows high prevalence of MSD among dental students in Saudi Arabia. Dental schools should encourage their students to apply principles of ergonomics, and the use of dental loupes during clinical sessions. Adding stretching classes to the curriculum and preparing an easily accessible school sports hall in the campus are arrangements that can minimize the further detrimental impact of MSD among students. Future studies should investigate the effect of psychosocial factors, such as stress and fatigue on the prevalence and severity of MSD.

### Supplementary Information

**Additional file 1.**

## Data Availability

The data can be provided by the corresponding author upon reasonable request.
