# Supplementary material for: Musculoskeletal disorders among dental students: a survey from Saudi Arabia
Source: BMC Oral Health. 2023 Oct 25;23:795. doi: 10.1186/s12903-023-03469-y (PMC10601255; doi:10.1186/s12903-023-03469-y)
Supplement: Supplementary file 1 — Additional file 1. [file 12903_2023_3469_MOESM1_ESM.docx]

Dear Student:

We are a group of researchers from college of Dentistry- Imam Abdulrahman Bin Faisal University investigating the prevalence of Musculoskeletal disorders and its risk factors among dental students in Saudi Arabia.  Musculoskeletal disorders are prevalent especially among dentists and dental students and can have serious consequences on one’s general health and career. We hope that the results of this study can help in identifying risk factors and risk groups and guide decision makers in setting preventive measures within dental schools.

You are kindly invited to participate in the study by filling out the survey which will not take more than 10 minutes to complete and share it with dental students in your college and other dental colleges within KSA.

Please fill out the survey only if 1) you are currently a dental student 2) you have no medical condition or not currently under any treatment.

Your participation is voluntary, and your information will remain confidential and will only be used for research purposes. You can quit the survey at any time, once you click the submit button your responses cannot be retrieved. Choosing to proceed with the survey will be considered as a consent to participate in the study.
 
For inquiries, please contact this email: [Msdresearch.sa@gmail.com](mailto:Msdresearch.sa@gmail.com)


Thank you for answering and sharing.

**Demographic data**

1. **Age**
2. **Gender**

- Male
- Female

1. **Region**

- Eastern
- Central
- Western
- Northern
- Southern

1. **Study year**

- Third year
- Fourth year
- Fifth year
- Sixth year
- Intern

1. **School**

- Public
- Private

1. **Type of practice**

- Clinical
- Pre- Clinical
- Both

1. **Height:**
2. **Weight:**

**Daily habits and lifestyle**

1. **Dominant hand**

- Right-hand
- Left-hand

1. **Is there a prepared clinic for left-handed students in your dental school?**

- Yes
- No
- I do not know

1. **Practice hours**

- Daily
- Weekly

1. **Average time of clinical\pre-clinical activities per day:**
   - 2-4 hours
   - 5-7 hours
   - 8-10 hours
2. **Which session do you feel more exhausted in?**

- Endodontics
- Restorative
- Fixed prosthodontics
- Removable prosthodontics
- Periodontics
- Pedodontics
- Oral maxillofacial surgery
- Orthodontics
- Oral maxillofacial Radiology

1. **Do you use dental magnification loupes?**

- Yes
- No

1. **Do you choose appropriate gloves size carefully?**

- Yes
- No
- I don’t care

1. **Which of the following do you practice? (Choose all that apply)**

- Workout
- Walking
- Yoga
- Meditation
- None of the above

1. **For how many hours per day do you use the electronic device?**

- 2-4h
- 4-6h
- 6-8h
- More than 8

1. **How many hours do you sleep on an average daily?**

- Less than 6
- More than 6

1. **How do you prefer to work?**

- Standing
- Sitting
- Both

1. **Do you believe that the pain affects your daily life?**

- Yes
- No
- Not applicable

**MSD questionnaire**

1. **Have you had Musculo- Skeletal pain during the last 12 months?**

- Yes
- No

1. **If yes, please specify the body parts with pain (If multiple symptoms please note):**

|  | Yes | No | **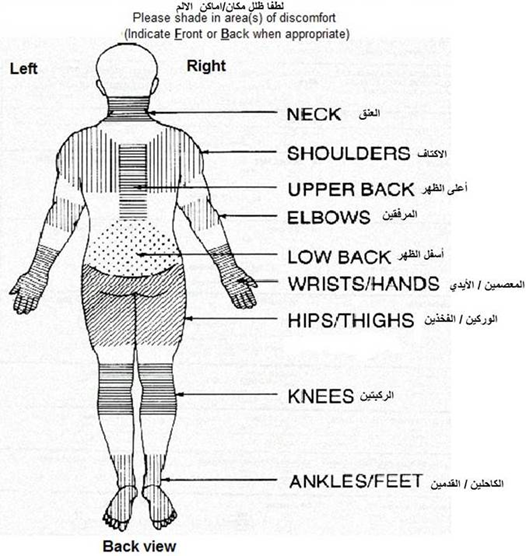** |
| --- | --- | --- | --- |
| 1. **Neck** |  |  |  |
| 1. **Shoulders** |  |  |  |
| 1. **Elbows** |  |  |  |
| 1. **Wrists and hands** |  |  |  |
| 1. **Upper Back** |  |  |  |
| 1. **Lower Back** |  |  |  |
| 1. **Hips\ Thighs** |  |  |  |
| 1. **Knees** |  |  |  |
| 1. **Ankles\ Feet** |  |  |  |
| 1. **Not applicable** |  |  |  |

**Management**

1. **How do you manage the pain?**

- Pain killers
- Hot bath
- Cupping therapy
- Not Applicable
- Other (please specify)

1. **Do you think working with an assistant will improve your condition?**

- Yes
- No
- Not applicable

1. **Do you believe you give sufficient attention to your health?**

- Yes
- No
